# Editorial: New concepts, advances, and future trends in clinical research on eye diseases

**DOI:** 10.3389/fmed.2026.1960305

**Published:** 2026-09-25

**Authors:** Weihua Yang, Huihui Fang, Qi Dai, Andrzej Grzybowski

**Affiliations:** 1 Shenzhen Eye Hospital, Shenzhen Eye Medical Center, Southern Medical University, Shenzhen, China; 2 Pazhou Lab, Guangzhou, China; 3 Eye Hospital, Wenzhou Medical University, Wenzhou, China; 4 Department of Ophthalmology, University of Warmia and Mazury, Olsztyn, Poland

**Keywords:** advances, artificial intelligence, clinical research, eye diseases, future trends, new concepts

Ocular diseases remain major causes of visual impairment and blindness worldwide, imposing substantial personal, social, and economic burdens (1). At the same time, ophthalmology is undergoing a profound transformation (2). Advances in ophthalmic imaging, disease mechanisms and signaling pathways, artificial intelligence (AI), and digital health are reshaping the future of ophthalmic research. Contemporary ophthalmic research is therefore moving beyond qualitative description toward quantitative characterization, mechanistic understanding, individualized risk assessment, and prediction of disease trajectories (3).

Several conceptual transitions characterize this evolution. Disease characterization is expanding from morphology alone toward functional, molecular, and systemic dimensions. Moreover, the eye is being recognized not only as an isolated organ but also as a potential window into systemic health (4). Ultimately, the central challenge is to translate increasingly complex and heterogeneous data into support for clinical research and treatment.

The Research Topic New Concepts, Advances, and Future Trends in Clinical Research on Eye Diseases brings together a broad and representative body of work spanning clinical research, technological innovation, and translational investigation. Seventy-nine articles, including original research, reviews, and case reports, were accepted. Rather than providing an exhaustive account of the entire Research Topic, this editorial highlights some representative research that illustrates the major themes and advances emerging across the Research Topic. These contributions can be broadly organized into six themes: Ophthalmic Imaging and Visual Function, Disease Biomarkers and Precision Medicine, Disease Progression and Predictive Modeling, Artificial Intelligence and Intelligent Ophthalmology, Clinical and Surgical Innovation, and Ophthalmic Epidemiology and Global Eye Health. Taken together, these studies illustrate a common trajectory in contemporary ophthalmology, which integrates precise measurement with biological insight and computational analysis.

The Figure 1 illustrates the contributing studies are distributed across six major themes: ophthalmic imaging and visual function, disease biomarkers and precision medicine, disease progression and predictive modeling, artificial intelligence and intelligent ophthalmology, clinical and surgical innovation, and ophthalmic epidemiology and global eye health.

**Figure 1:**
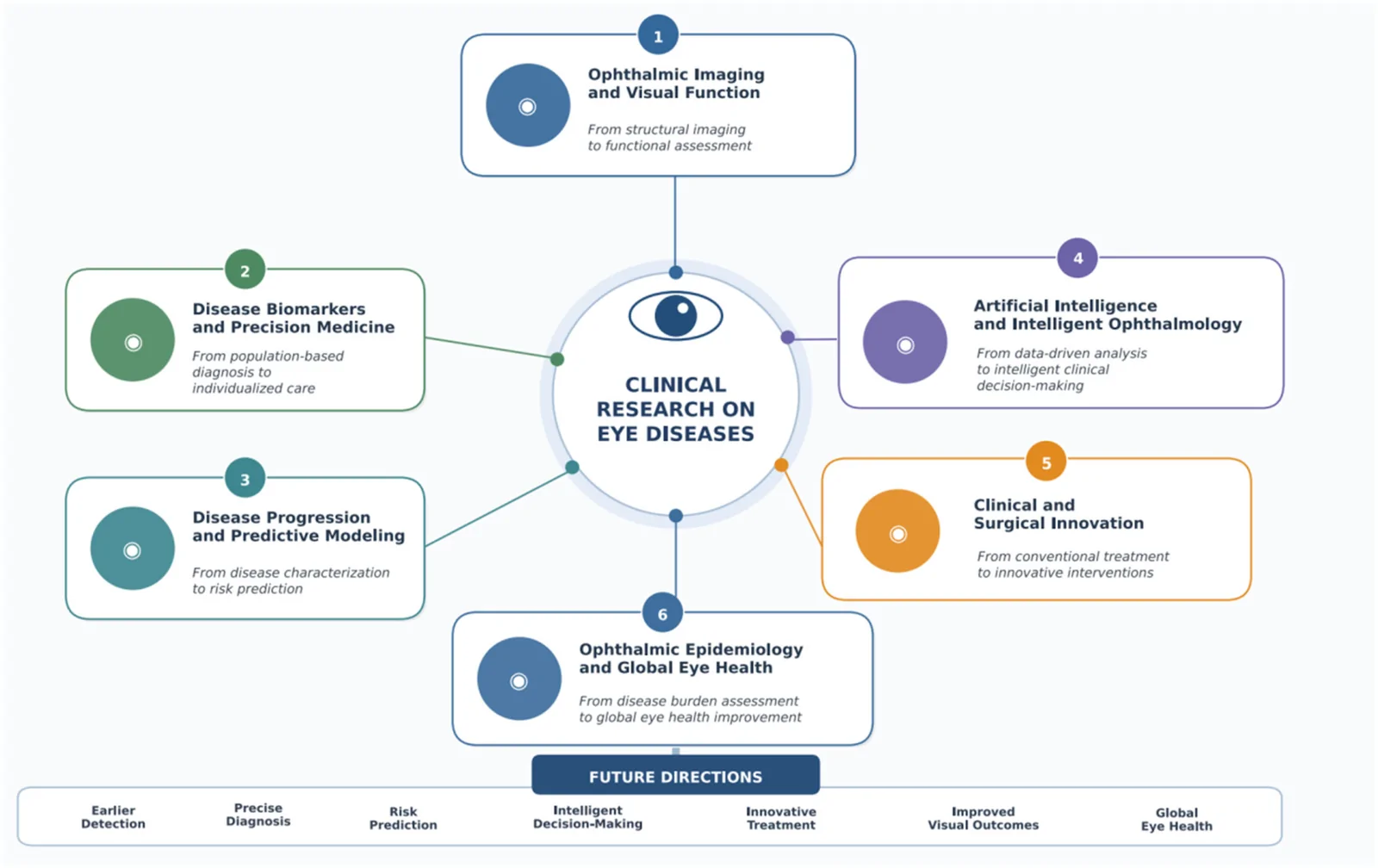
Schematic overview nnof the six major themes and representative contributions in the research topic.

## Ophthalmic imaging and visual function

Ophthalmic imaging has evolved from a primary visualization tool into a quantitative platform of anatomical structure characterization (5). Several studies in this research topic demonstrate the value of quantitative imaging in linking structural or vascular changes with functional impairment. Zhang et al. investigated macular vascular density in patients with chronic primary angle-closure glaucoma and showed that macular microvascular alterations were associated with visual field damage. Similarly, Tang et al. examined interocular differences in choroidal thickness and circulation in adults with anisomyopia, providing evidence that greater myopic severity may be associated with more pronounced alterations in choroidal structure and function. Moreover, the retina provides a unique and directly accessible view of the human microvasculature, and may offer insights into systemic disease. Liang et al. explored retinal structural and vascular alterations in individuals with suboptimal cardiovascular-kidney-metabolic health using OCTA, highlighting the potential of ocular imaging as a noninvasive approach for identifying early systemic abnormalities. Together, these studies illustrate a broader shift from simply visualizing ocular abnormalities toward identifying quantitative imaging phenotypes relevant to disease severity, progression, and systemic health.

### Disease biomarkers and precision medicine

The combination of imaging with biological mechanisms promotes precision medicine, as molecular and systemic biomarkers may provide complementary information regarding the pathways underlying disease development and progression (6, 7). Chen et al. investigated the association between the ratio of non-high-density lipoprotein cholesterol to high-density lipoprotein cholesterol (NHHR) and age-related macular degeneration (AMD) through the National Health and Nutrition Examination Survey and then validated their findings using external datasets. Their findings suggest that NHHR may contribute to risk stratification for AMD and further support the relevance of metabolic factors in ocular disease. Ma et al. explored the diagnostic value of aqueous humor Syndecan-1 and Syndecan-4 in diabetic retinopathy in conjunction with OCTA imaging, illustrating the complementary potential of molecular and imaging markers in understanding disease biology. Hu et al. combined dynamic amplitude of low-frequency fluctuation analysis with transcriptomic approaches to investigate potential neurophysiological mechanisms in primary angle-closure glaucoma, emphasizing that glaucoma may involve broader neural alterations beyond structural damage. These studies collectively suggest that future biomarker discovery will increasingly move toward multimodal approaches and integration.

### Disease progression and predictive modeling

The ability to anticipate disease behavior and treatment outcomes is central to precision ophthalmology (8). Lou et al. investigated the relationship between standardized echographic morphological characteristics and prognosis in uveal melanoma, demonstrating that tumor shape classification may provide independent prognostic information and clinical risk stratification. Similarly, Wang et al. evaluated treatment outcomes of intravitreal conbercept for chronic central serous chorioretinopathy with occult choroidal neovascularization and explored the prognostic relevance of lesion characteristics, illustrating the potential of multimodal imaging to support individualized prediction of therapeutic outcomes. Importantly, predictive research should not be interpreted solely as the development of statistical models. Its broader objective is to identify clinically actionable information that can support earlier intervention, improve patient stratification, and reduce unnecessary treatment burden. Achieving this goal will require rigorous external validation, assessment of clinical utility, and integration into real-world workflows.

### Artificial intelligence and intelligent ophthalmology

AI is becoming an increasingly important component of ophthalmic research. Its applications are expanding from automated image classification toward quantitative measurement, multimodal analysis, risk prediction, clinical decision support, and patient communication (9–11). He et al. evaluated the performance of several large language models in responding to questions related to optic neuritis, highlighting both the potential and current limitations of generative AI as a clinical information and consultation tool. Similarly, Wang et al. assessed the performance of ChatGPT in thyroid eye disease in image-based assessment and clinical consultation, illustrating the emerging role of vision language models in ophthalmic communication and decision support. Predictive modeling also represents an important application of machine learning. Wang et al. investigated the occurrence and progression of postoperative amblyopia in patients with congenital lens subluxation and developed a model for individualized risk prediction. Xiao et al. developed a predictive model for orthokeratology lens (Ortho-K) decentration after one month of wear, identifying the contribution of corneal morphological characteristics and age to Ortho-K stability. Although the applications are expanding, robust external validation, transparency assessment, prospective evaluation, and human supervision are essential before clinical adoption (12).

### Clinical and surgical innovation

Innovations in clinical management and surgical intervention are essential for improving safety, precision, and treatment efficiency (13). Yu et al. evaluated photorefractive keratectomy with an extended ablation zone for recurrent corneal erosion syndrome associated with refractive error, providing evidence for an alternative strategy that simultaneously addresses corneal pathology and refractive status. In primary open-angle glaucoma, Wang et al. reported preliminary outcomes of trabeculotome tunneling trabeculoplasty, demonstrating sustained intraocular pressure reduction and decreased medication requirements with a relatively low incidence of postoperative complications. Precision is equally relevant to laser treatment. Xie et al. evaluated the minimum effective laser power for subthreshold micropulse laser treatment in acute and chronic central serous chorioretinopathy, providing individualized treatment parameters to help balance therapeutic efficacy with tissue safety. In summary, clinical innovation makes it possible to better balance efficacy, safety, accessibility, and treatment burden.

### Ophthalmic epidemiology and global eye health

Population-based research remains essential for understanding the broader distribution and determinants of visual impairment (14). Wan et al. analyzed the global, regional, and national burden of cataract among adults aged 60 years and older from 1990 to 2021 using Global Burden of Disease 2021 data. Their findings emphasize the persistent and unequal burden of cataract-related visual impairment and reinforce the importance of strengthening access to effective eye health services. Similarly, Bai et al. examined global trends in eye cancer across 204 countries and territories, revealing substantial geographic disparities in incidence, mortality, and disability-adjusted life years. These findings highlight the need to improve diagnostic infrastructure and reduce inequalities in access to specialized care. Wu et al. investigated the association between the weight-adjusted waist index and AMD among adults aged 40 years and older in the United States. The observed nonlinear association further supports the potential relevance of systemic metabolic health to retinal disease. These studies demonstrate that improving global eye health will require the integration of epidemiological evidence, prevention strategies, health policy, and equitable implementation (15).

## Conclusion

Taken as a whole, the studies reflect a broader transformation in ophthalmic clinical research. A particularly important emerging direction is the integration of longitudinal multimodal data. Future approaches, including digital twin approaches and other data-driven models, may provide opportunities to combine imaging, clinical characteristics, biological information, and treatment responses into dynamic representations of individual patients. The next frontier of ophthalmic research will require closer alignment between technological innovation and rigorous clinical validation. Standardized data generation, multicenter and prospective studies, transparent and responsible AI governance, health-economic evaluation, and equitable implementation will all be critical. Only through such integration can advances in imaging, biological science, computational medicine, and clinical innovation be translated into measurable improvements in the prevention, diagnosis, monitoring, and treatment of eye diseases.
